# SNPs in the FCER1A Gene Region Show No Association with Allergic Rhinitis in a Han Chinese Population

**DOI:** 10.1371/journal.pone.0015792

**Published:** 2010-12-31

**Authors:** Yuan Zhang, Su Duan, Xiaoping Lin, Wei Zhang, Na Meng, Liping Zhao, Yan Zhao, Demin Han, Luo Zhang

**Affiliations:** 1 Key Laboratory of Otolaryngology, Head and Neck Surgery, Ministry of Education of China, Beijing Institute of Otorhinolaryngology, Beijing, People's Republic of China; 2 Department of Otolaryngology, Head and Neck Surgery, Beijing TongRen Hospital, Capital Medical University, Beijing, People's Republic of China; 3 Center of Allergy and Immunotherapy, The General Hospital of Shenyang Military Command, Shenyang, People's Republic of China; Ohio State University Medical Center, United States of America

## Abstract

**Background:**

Immunoglobulin E (IgE) is a central player in the allergic response, and raised total IgE levels are considered as an indicator of atopy or potential development of atopy. A recent genome-wide scan in a German population-based cohort of adults identified the gene encoding the alpha chain of the high affinity receptor for IgE (FCER1A) as a susceptibility locus influencing total serum IgE levels. The aim of this study was to investigate whether the polymorphisms in the FCER1A gene are associated with allergic rhinitis (AR) in a Han Chinese population.

**Methodology/Principal Findings:**

A population of 378 patients with AR and 288 healthy controls was studied. Precise phenotyping of patients was accomplished by means of a questionnaire and clinical examination. Blood was drawn for DNA extraction and total serum immunoglobulin E (IgE) measurement. A total of 16 single nucleotide polymorphisms (SNPs) in FCER1A were selected and individually genotyped. None of the SNPs in the FCER1A showed an association with AR. Similarly, the lack of association was also evident in subgroup analysis for the presence of different allergen sensitivities. None of the selected SNPs in FCER1A was associated with total IgE level.

**Conclusions:**

Although FCER1A presents itself as a good candidate for contributing to total serum IgE, this study failed to find an association between SNPs in the FCER1A gene region and IgE level or AR susceptibility.

## Introduction

Allergic rhinitis (AR) is an inflammatory disease of the nasal mucosa induced by an immunoglobulin E (IgE)-mediated reaction in allergen-sensitized subjects. It is a disease of high prevalence, especially in industrialized countries, and has exhibited a fairly rapid upward trend [1]. Recent data on self-reported AR in the centre of cities across mainland China demonstrated a prevalence of 8.7%–24.1% [2]. This high prevalence translates into a considerable cost to society in terms of overall healthcare use and the quality of life of those with moderate-to-severe disease. The reasons for these trends remain unclear but probably reflect environmental influences on genetic predisposition.

High total serum IgE levels are closely correlated with the clinical expression and severity of asthma and allergy [3], [4]. The regulation of serum IgE production is largely influenced by familial determinants, and both pedigree- and twin-based studies provide evidence of a strong genetic contribution to the variability of total IgE levels [5], [6]. Genetic susceptibility to IgE-responsiveness is likely to be caused by a pattern of polymorphisms in multiple genes regulating immunological responses [7], but so far only a very few loci have been established consistently and robustly [8], [9]. Very recently, a genome-wide scan in a German population-based cohort of adults identified the gene encoding the alpha chain of the high affinity receptor for IgE (FCER1A) on chromosome 1 as a susceptibility locus which was associated with total serum IgE levels [10]. Moreover, Chen *et al.* also found that a common variant in FCER1A was associated with total serum IgE levels found in cord blood and up to the first six years of life, and this was demonstrated to be independent of environmental stimuli [11].

We hypothesized that FCER1A could be a strong candidate gene for influencing total serum IgE level and that single nucleotide polymorphisms (SNPs) in the FCER1A gene region may influence the risk of developing AR. Therefore, in an attempt to identify whether and how polymorphisms in the FCER1A gene are associated with AR, we conducted a population-based case-control association analysis to assess the risk of AR conferred by SNPs in the FCER1A gene region in a Han Chinese cohort.

## Results

### Population characteristics

The characteristics of the study population are shown in Table 1. Age and gender were well-balanced between cases and controls. The cohort of 378 AR patients had a mean age of 27 years and consisted of more men (60.1%) than women (39.9%), while the 288 control individuals had a mean age of 37 years, with a similar component of more men (56.1%) than women (43.9%). The mean total serum IgE measurements for the case and control groups were 292.3±529.8 and 58.2±111.7 IU/ml respectively. Patients were diagnosed as having AR by combining the skin test and serum specific IgE data with the allergen-specific case history and physical nasal examination. 83.1% of individuals demonstrated perennial nasal symptoms while the remainder reported seasonally-related reactions. When subjects were classified according to the serum allergen-specific IgE category, 216 (56.6%), 64 (16.9%) and 100 (26.5%) were allergic to house dust mites (HDM), pollens and mixed allergens respectively. We defined mixed allergen allergy to be when an individual showed positive responses to two or more allergens in the skin test or serum examination.

**Table 1:** Demographic characteristics of the study population.

Characteristic	AR cases (n = 378)	Controls (n = 288)
Age Mean (Range) (years)	26.9±14.8 (2–71)	36.3±15.2 (3–78)
Sex, M/F, No. (%)	227 (60.1)/151 (39.9)	161 (56.1)/126 (43.9)
Total IgE Mean (Range), kU/l	292.3±529.8 (6.94–5000)	58.2±111.7 (2–906)
Perennial/seasonal, No.(%)	314 (83.1)/64 (16.9)	/
Allergen category, No. (%)		
House dust mite	214 (56.6)	/
Pollens	64 (16.9)	/
Mixed allergens	100 (26.5)	/

“/”: no results.

### Single marker association analysis of FCER1A SNPs with AR susceptibility

**Figure 1:**
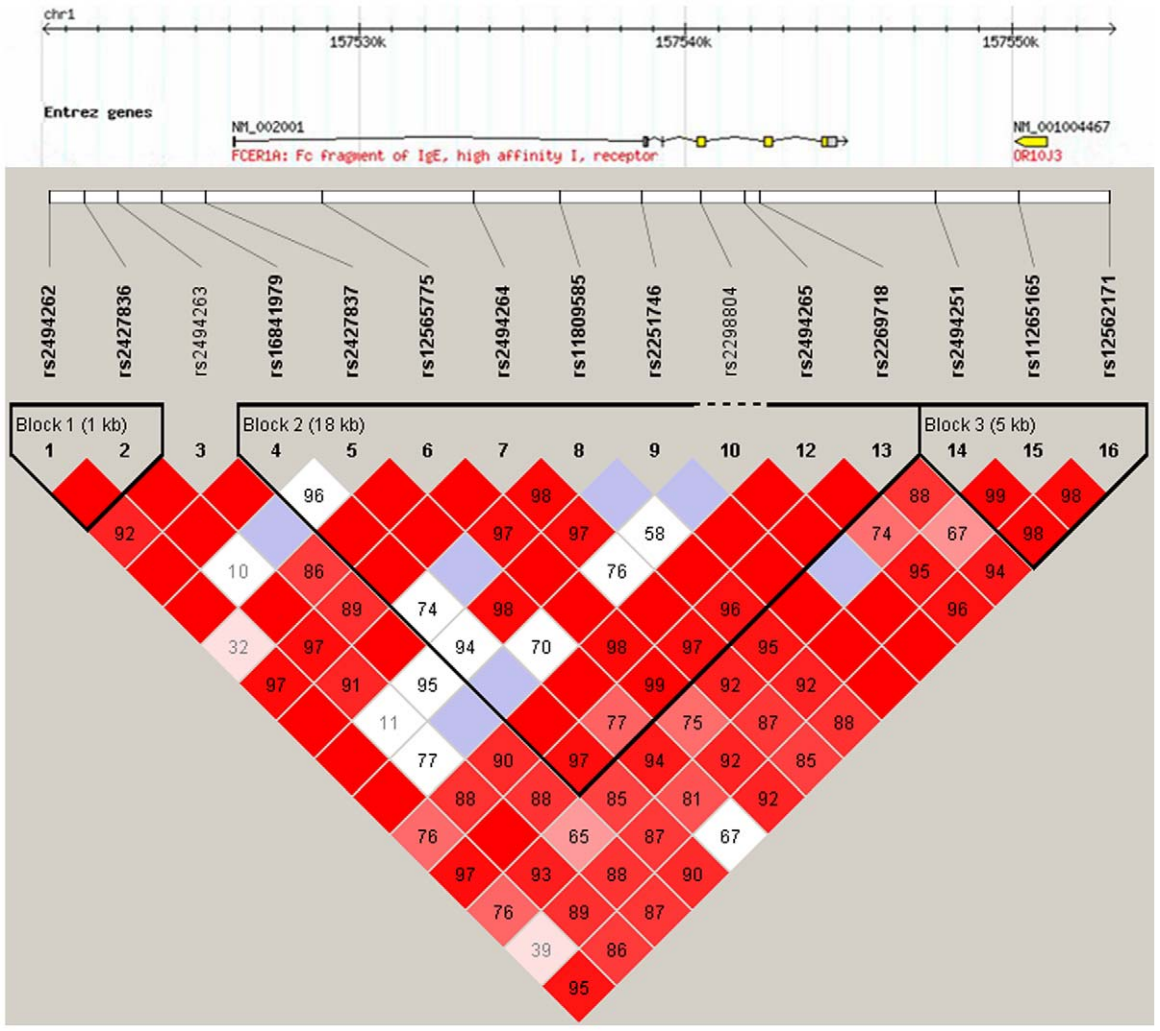
Pairwise Linkage Disequilibrium (LD) between tagging SNPs for the FCER1A gene. The LD plots were generated by Haploview 4.1. The white horizontal bar below the info track illustrates the location of SNPs on a physical scale. The value within each diamond represents the pairwise correlation between tagging SNPs defined by the upper left and the upper right sides of the diamond. Shading represents the magnitude and significance of pairwise LD, with a red-to-white gradient reflecting higher to lower LD values.

**Figure 2:**
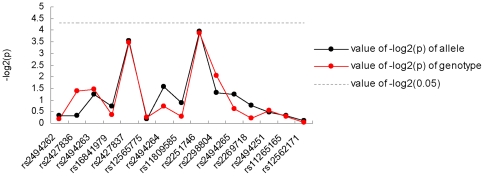
Significance of each tagging SNP. The *x* axis shows the genomic position, and the *y* axis shows the negative base-10 logarithm of the *P* values for each allele and genotype of each SNP. Lines are drawn between points only for the sake of clarity, as the disease association between markers is not linear.

We genotyped SNPs in the FCER1A gene, tagging three blocks (Figure 1). One SNP, rs2298805 (exact *P* = 0.0071) was not in Hardy-Weinberg equilibrium (HWE) in controls and was excluded from further analysis. The analysis of allele frequencies for the remaining 15 SNPs tested, shown in Table 2, revealed no significant associations (*P*<0.05) with an AR outcome in the FCER1A gene. Meanwhile, results for the analysis of association between the related genotypes of each SNP and AR susceptibility were also obtained, again indicating a lack of evident association (Table 2). Although none of the SNPs showed evidence of association, rs2251746 (*P*_allele_ = 0.0659; *P*_genotype_ = 0.0688) and rs2427837 (*P*_allele_ = 0.0865; *P*_genotype_ = 0.0904) exhibited relatively lower *P* values among the 15 selected SNPs (Figure 2). Likewise, stratifying the AR cohort and controls by the presence of sensitivity to different allergens failed to detect any evidence of association between the genotyped SNP markers and AR (Table S1). In addition, none of the 15 SNPs in the FCER1A gene was associated with serum total IgE level (Table 3).

**Table 2:** Allele and genotype frequencies and AR susceptibility.

SNP	Minor allele	Case, Control Frequencies	Allele *P*-value	Genotype *P*-value
rs2494262(A/C)	C	0.2715, 0.2780	0.7961	0.8875
rs2427836(C/T)	C	0.1953, 0.1895	0.796	0.3782
rs2494263(A/G)	G	0.1619, 0.1448	0.4242	0.3640
rs16841979(C/G)	C	0.0822, 0.0743	0.6045	0.7709
rs2427837(A/G)	A	0.0417, 0.0629	0.0865	0.0904
rs12565775(A/C)	A	0.4432, 0.4477	0.8743	0.8446
rs2494264(A/T)	A	0.2201, 0.2428	0.3386	0.5990
rs11809585(A/T)	T	0.0880, 0.0978	0.5485	0.8247
rs2251746(C/T)	C	0.0403, 0.0629	0.0659	0.0688
rs2298804(A/G)	G	0.0376, 0.0290	0.3999	0.2440
rs2494265(A/T)	A	0.2028, 0.1848	0.4221	0.6440
rs2269718(A/G)	A	0.4391, 0.4239	0.5887	0.8591
rs2494251(A/G)	A	0.2799, 0.2709	0.7213	0.6810
rs11265165 (C/T)	C	0.4319, 0.4388	0.8052	0.8185
rs12562171(C/T)	C	0.3663, 0.3687	0.9295	0.9815

**Table 3:** Association between FCER1A SNPs and IgE levels.

SNP	*P* for Levene Statistic	F	*P*-value
rs2494262	<0.001	1.653*	0.438*
rs2427836	0.294	0.695	0.556
rs2494263	0.087	1.181	0.317
rs16841979	0.824	0.238	0.917
rs2427837	0.632	0.185	0.906
rs12565775	0.771	0.149	0.931
rs2494264	0.219	0.743	0.563
rs11809585	0.669	1.081	0.366
rs2251746	0.651	0.157	0.925
rs2298804	0.639	0.254	0.907
rs2494265	0.218	0.550	0.699
rs2269718	0.054	0.832	0.505
rs2494251	0.038	1.641*	0.44*
rs11265165	0.243	0.498	0.684
rs12562171	0.009	1.137*	0.566*

“*”: results using Kruskal-Wallis ANOVA.

### LD and haplotype analysis

To assess the extent of linkage disequilibrium (LD) across the FCER1A gene, we calculated D′ values. Some of the pair-wise D′ values in the FCER1A gene were close to 1 among all SNPs, indicating very strong LD. As the SNPs were in 3 different LD blocks, the haplotypes of the markers of the FCER1A gene were analyzed separately. Among all the haplotypes which were predicted, only 15 had a frequency above 1%. Again, no significant difference was detected between AR cases and healthy controls (Table 4).

**Table 4:** Haplotype frequencies and AR susceptibility.

Haplotype	Case Fr	Control Fr	df	Chi Square	P-Value
Block 1					
AT	0.533	0.532	1	0.001	0.9788
CT	0.271	0.278	1	0.067	0.7961
AC	0.195	0.190	1	0.067	0.796
Block 2					
GGCTATTA	0.358	0.346	1	0.189	0.6635
GGATATAG	0.200	0.181	1	0.729	0.3932
GGCTATTG	0.117	0.127	1	0.312	0.5764
GGAATTTG	0.084	0.098	1	0.759	0.3836
GGAAATTG	0.093	0.081	1	0.572	0.4496
CGCTATTA	0.079	0.074	1	0.135	0.7136
GAAAACTG	0.040	0.062	1	3.029	0.0818
GGATATTG	0.021	0.024	1	0.11	0.7403
Block 3					
GCC	0.362	0.361	1	0	0.9939
GTT	0.285	0.286	1	0.002	0.9624
ATT	0.280	0.268	1	0.215	0.6432
GCT	0.070	0.076	1	0.185	0.6675

*df*: degree of freedom.

## Discussion

Our study aimed to evaluate the contribution of single nucleotide polymorphisms (SNPs) in the FCER1A gene region to allergic rhinitis (AR) susceptibility in a Chinese population-based case-control association analysis. The impact of common FCER1A SNPs on AR susceptibility was first measured in the Chinese cohort. Our data shows no associations between SNPs in the FCER1A gene region and AR.

Allergic sensitization is defined by production of immunoglobulin E (IgE) against environmental antigens such as house dust mite, grass pollen, and animal proteins and can lead to diseases that include asthma, rhinitis and atopic dermatitis [12]. A key feature of these diseases is an IgE-mediated aberrant immune responsiveness to otherwise harmless environmental allergens, with the majority of patients exhibiting increased total and allergen-specific IgE levels [13]. Numerous metabolic networks can contribute to IgE production, but their complex interactions are not well understood at present [14].

FCER1A is an important immunity-related gene that encodes the ligand-binding subunit of the high-affinity IgE receptor (FceRI), the a-chain (FceRIa) [15]. Thus, recent studies have focused not only on human FCER1A gene variability [16]–[22], but also on polymorphisms of its counterparts in animals [[23].[24]]. Hasegawa first reported that a significant portion of nonallergic individuals has heterozygous −66T/C genotype of rs2251746 in FCER1A gene, while most of allergic individuals have homozygous −66T/T genotype in Japanese population in 2003 [17]. Moreover, in a recent population-based GWAS,,a functional promoter variant affecting FCER1A expression (rs2251746) and a tightly-linked variant (rs2427837) were found to be highly associated with total serum IgE levels, a finding that was replicated in more than 10 000 individuals from four independent population-based cohorts [10]. In a subsequent independent replication study on two German birth cohorts, they showed that these variants were strongly associated with basal IgE production independently of environmental stimuli and at different stages of life, operating as early as during the fetal period [11]. Very recently, Mahachie *et al.* reported that three strongly-correlated FCER1A polymorphisms were significantly associated with total and specific IgE levels as well as with allergic sensitization, suggesting that FCER1A variants, either alone or in combination, influence IgE levels and act synergistically to influence eczema risk [25].

The extent of the impact of polymorphisms on FCER1A function and properties is under investigation. Many atopy-associated SNPs within the promoter of FCER1A have been reported to determine transcriptional activity and subsequently receptor expression. In particular, FcεRIα −344(−315)1 C>T (rs2427827) polymorphism was found to be associated with aspirin-induced urticaria [21] and with total serum IgE levels in different groups of allergic subjects [16], [18], [19], [21]. Notably, in a previous study with Japanese individuals it could be shown that the minor allele of the polymorphism FCER1A proximal promoter −95(−66)1 T>C (rs2251746) which is close genomic neighbor of rs2427827 is associated with higher FCER1A expression through enhanced GATA-1 binding [17]. Both of these frequent polymorphisms were demonstrated to strongly affect FCER1A expression in mast cells and/or basophils in an additive manner [17], [21], [22], thus providing a partial mechanistic background for the genetic associations described above. In addition, the frequent −18483A>C (rs2494262) polymorphism was also found to be associated with total serum IgE levels in allergic subjects [18]–[20]. Very recently, Potaczek et al found that we confirmed preferential binding of the YY1 transcription factor to the −18483C allele, resulting in lower transcriptional activity when compared with the −18483A allele [26].

Total IgE is a strongly heritable quantitative trait [27], [28]. In contrast to the considerable genetic and phenotypic heterogeneity at the level of allergic disease, total IgE represents a more defined and quantifiable endophenotype. Although not without controversy, it is commonly thought that it is useful to study such low-level physiological traits associated with the higher-order diseases of interest, because they probably involve fewer genes and fewer interacting levels, and eventually provide simpler clues to an enhanced functional understanding of the genetics of the more complex diseases [29], [30]. Interestingly, although in this study we did not detect associations between FCER1A variants and total IgE level or AR, rs2251746 and rs2427837 exhibited the highest *P* values for associations among the 15 selected SNPs in our data. This is similar to Weidinger's GWAS, which identified rs2251746 and rs2427837 as being strongly associated with total IgE levels in all cohorts with significant P values and found that these two SNPs were in a complete LD.

Furthermore, it is important to note that the lack of association in this study does not completely rule out FCER1A as an AR candidate gene. The statistical power of sample size in this study was only 77.85% as regards the association analysis. However, it could be that SNPs in the FCER1A gene region confer a smaller risk of developing AR. Future efforts to identify FCER1A SNPs carrying a smaller FCER1A risk will require a larger sample size than has been used here. Moreover, as with many other complex disorders, AR is thought to be the result of a complicated network of numerous susceptibility loci, many of which exert additive or synergistic effects, but have only a small role when considered in isolation [31], [32]. Among other reasons, failure to identify and replicate individual genetic risk factors for complex diseases has been attributed to epistasis obscuring the effect of single loci [33], [34]. Designing suitable analytical strategies to identify multi-SNP interactions in addition to factors that exhibit an independent effect is also one of the main challenges for GWASs. However, it is important to note that based on the current study, a particular FCER1A variant alone does not show a significant association with the development of AR. Further studies including gene-gene and gene-environment interactions in AR cohorts are needed to clarify the impact of FCER1A on atopic disease.

In conclusion, currently there is no genetic evidence to suggest that polymorphisms in the FCER1A gene region confer susceptibility to AR or IgE levels in a Han Chinese population.

## Materials and Methods

### Study subjects

Three hundred and seventy-eight individuals (227 males and 151 females) with AR were prospectively recruited from the rhinology clinic and ward of Beijing TongRen Hospital and the Allergy and Immunotherapy Center of The General Hospital of Shenyang Military Command from February 2008 to July 2009. Individuals with accompanying allergic diseases such as asthma and atopic dermatitis were excluded from the study. A total of 288 controls who were healthy volunteers were recruited from an ethnically-similar local population to determine similar background population allele frequencies. All subjects were of Han Chinese ethnic origin and all from the northern region of China. The study was approved by the Ethics Committees of Beijing TongRen Hospital and The General Hospital of Shenyang Military Command, and written informed consent was obtained from all participants.

Patients were diagnosed as having AR if they tested positive for all three of the following criteria from the ARIA (Allergic Rhinitis and its Impact on Asthma, 2008) [35] guidelines. 1) persistent or discontinuous symptoms of anterior rhinorrhea, continuous sneezing, nasal obstruction and itching, 2) local nasal cavity signs, including discolored and edematous mucosa and watery drainage in the common or middle nasal meatus and 3) positive serum antigen-specific IgE as measured by the ImmunoCAP 100 system (Pharmacia, Uppsala, Sweden) and positive antigen skin prick test (SPT) (Allergopharma, Reinbeck, Germany). The antigens included house dust mite (HDM) (*Dermataphagoides farinae and D. pteronyssinus*); seasonal grass pollens (Giant Ragweed; Mugwort; Lamb's quarters; *Humulus*; *Chenopodium album*, etc.); animal hair (especially dog and cat); molds (indoor and outdoor mustiness or of floriculture), and cockroach. Subjects were considered to be sensitive to allergens if the measurement of serum IgE was equal to or above 0.35 kU/l. A positive SPT result was defined as a wheal greater than or equal to one half of the diameter of the histamine control and at least 3 mm larger than the diameter of the negative control [36]. Serological and skin testing were performed by specialist technicians and nurses respectively, while the AR diagnoses were made by clinical rhinologists. The controls presented no clinical features, local nasal cavity signs, family history of AR and showed negative of serum antigen-specific IgE phadiatop determination.

### Selection of polymorphisms in the human FCER1A gene

The International Haplotype Mapping (HapMap) (www.hapmap.org) SNP databases were used to select SNPs in the FCER1A gene region. The screened region was extended 10 kilobases upstream of the annotated transcription start site and downstream at the end of the last FCER1A exon. The SNPs were selected to extract the most genetic information based on CHB haplotype data using the HAPMAP database (Hapmap Data Rel 27 Phase II+III, Feb09) [37]. From this dataset, 29 SNPs in the FCER1A gene region were selected using a pairwise tagging algorithm implemented in Haploview software version 4.1 [38]. In addition, when we set the Hardy-Weinberg *p* value cutoff, minor allele frequency and r^2^ thresholds at 0.01, 0.05 and 0.8, respectively, the linkage disequilibrium (LD) pattern for the FCER1A gene in our population exhibited strong LD in several groups of SNPs, indicating that the SNPs in each group represent a common region. Consequently, we choose rs2494265, rs12565775, rs2269718, rs2494264, rs11265165, rs2494262, rs12562171, rs2494263 rs11809585, rs16841979, rs2298804, rs2298805, rs2427836 and rs2494251 to represent the entire 29 loci for eventual genotyping. Two additional SNPs (rs2251746, rs2427837) from a previous study were also included [10]. Therefore, 16 SNPs constituted the selection set to be genotyped in our patient and control groups.

### Single nucleotide polymorphism genotyping

DNA was isolated from peripheral blood leukocytes and collected in EDTA-treated tubes, using the DNA Isolation Kit for Mammalian Blood(Roche, Indianapolis, USA). Isolated DNA from blood was stored at 4°C prior to use. The majority of the selected SNP genotyping was performed using the Sequenom MassARRAY iPLEX Gold platform (Sequenom, San Diego, California) according to the manufacturer's instructions. The polymerase chain reaction (PCR) and extension primers were designed using MassARRAY Assay Design 3.1 software (for details see Table S2). One SNP (rs2494863), which was evaluated by preliminary testing as unsuitable for genotyping through the MassArray approach was identified by direct sequencing of the PCR products of genomic DNA. Genotyping was performed without knowledge of the case or control status. A 10% random sample was tested in duplicate by different persons, and the reproducibility was 100%.

### Statistical analyses

Hardy-Weinberg equilibrium (HWE) of each SNP was assessed in controls using a chi–square test with one degree of freedom using Haploview 4.1. A threshold of *P*<0.05 was considered to indicate deviation from HWE. SPSS software version 13.0 was used to determine association. Chi-square tests were performed to determine whether an association existed between cases and controls. Haplotype association analysis was performed using SHEsis, a web-based software platform that allowed the estimation of haplotype frequencies. Associations with susceptibility to AR were tested by calculating odds ratios (OR) with asymptotic 95% confidence intervals (95% CI), and corrected *P* values less than 0.05 were considered statistically significant. Furthermore, subanalysis restricted to the presence of different allergens - house dust mite, pollens and mixed allergens - was also performed to examine whether the effect of associations within the population differed among the subgroups. Associations between genotype and IgE levels for all AR and control subjects were assessed using an ANOVA test. Homogeneity of variance test was made using Levene test firstly. A threshold of *P* for Levene Statistic <0.05 was considered to indicate a non-normal distribution of IgE level. Kruskal-Wallis ANOVA was used for association of FCER1A SNPs with non-normal distribution IgE levels. The statistical power for the present study was calculated using G*Power 2 software (http://www.psycho.uni-duesseldorf.de/aap/projects/gpower/). When the parameter was set on 0.2 which represented the effect of the gene was minor, the power of the present collected sample size as regarding the association study was evaluated.

## Supporting Information

Table S1 **Allergen‐specific association analyses of SNPs in FCER1A and AR**.
(DOC)

Table S2 **Details of the primers used in the screening of SNPs by MassArray and PCR direct sequencing**.
(DOC)
