# Supplementary material for: SNPs in the FCER1A Gene Region Show No Association with Allergic Rhinitis in a Han Chinese Population
Source: PLoS One. 2010 Dec 31;5(12):e15792. doi: 10.1371/journal.pone.0015792 (PMC3013135; doi:10.1371/journal.pone.0015792)
Supplement: Table S1 — Allergen‐specific association analyses of SNPs in FCER1A and AR. (DOC) [file pone.0015792.s001.doc]

| SNP Locus (1/2) | House dust mite | | | | |  | Pollens | | | | |  | Mixed allergens | | | | |
| --- | --- | --- | --- | --- | --- | --- | --- | --- | --- | --- | --- | --- | --- | --- | --- | --- | --- |
|  | Control Frq | | Case Frq | | *P*-value |  | Control Frq | | Case Frq | | *P*-value |  | Control Frq | | Case Frq | | *P*-value |
|  | 1 | 2 | 1 | 2 |  |  | 1 | 2 | 1 | 2 |  |  | 1 | 2 | 1 | 2 |  |
| rs2494262 (A/C) | 0.7220 | 0.2780 | 0.7083 | 0.2917 | 0.664 |  | 0.7220 | 0.2780 | 0.7419 | 0.2581 | 0.739 |  | 0.7220 | 0.2780 | 0.7632 | 0.2368 | 0.297 |
| rs2427836 (C/T) | 0.1895 | 0.8105 | 0.2034 | 0.7966 | 0.622 |  | 0.1895 | 0.8105 | 0.2339 | 0.7661 | 0.263 |  | 0.1895 | 0.8105 | 0.1526 | 0.8474 | 0.275 |
| rs2494263(A/G) | 0.8528 | 0.1472 | 0.8239 | 0.1761 | 0.260 |  | 0.8528 | 0.1472 | 0.8679 | 0.1321 | 0.764 |  | 0.8528 | 0.1472 | 0.8587 | 0.1413 | 0.904 |
| rs16841979 (C/G) | 0.0743 | 0.9257 | 0.0837 | 0.9163 | 0.627 |  | 0.0743 | 0.9257 | 0.0806 | 0.9194 | 0.709 |  | 0.0743 | 0.9257 | 0.0789 | 0.9211 | 0.874 |
| rs2427837 (A/G) | 0.0629 | 0.9371 | 0.0369 | 0.9630 | 0.079 |  | 0.0629 | 0.9371 | 0.0403 | 0.9597 | 0.404 |  | 0.0629 | 0.9371 | 0.0526 | 0.9474 | 0.725 |
| rs12565775 (A/C) | 0.4460 | 0.5540 | 0.4559 | 0.5441 | 0.844 |  | 0.4460 | 0.5540 | 0.4839 | 0.5161 | 0.486 |  | 0.4460 | 0.5540 | 0.3895 | 0.6105 | 0.175 |
| rs2494264 (A/T) | 0.2428 | 0.7572 | 0.2291 | 0.7709 | 0.646 |  | 0.2428 | 0.7572 | 0.2500 | 0.7500 | 0.908 |  | 0.2428 | 0.7572 | 0.1809 | 0.8191 | 0.087 |
| rs11809585 (A/T) | 0.9022 | 0.0978 | 0.9080 | 0.0920 | 0.824 |  | 0.9022 | 0.0978 | 0.8871 | 0.1129 | 0.621 |  | 0.9022 | 0.0978 | 0.9368 | 0.0632 | 0.183 |
| rs2251746 (C/T) | 0.0629 | 0.9371 | 0.0345 | 0.9655 | 0.054 |  | 0.0629 | 0.9371 | 0.0403 | 0.9597 | 0.404 |  | 0.0629 | 0.9371 | 0.0526 | 0.9474 | 0.725 |
| rs2298804 (A/G) | 0.9710 | 0.0290 | 0.9629 | 0.0371 | 0.580 |  | 0.9710 | 0.0290 | 0.9839 | 0.0161 | 0.550 |  | 0.9710 | 0.0290 | 0.9474 | 0.0526 | 0.167 |
| rs2494265 (A/T) | 0.1848 | 0.8152 | 0.2118 | 0.7882 | 0.323 |  | 0.1848 | 0.8152 | 0.1935 | 0.8065 | 0.800 |  | 0.1848 | 0.8152 | 0.1895 | 0.8105 | 0.914 |
| rs2269718 (A/G) | 0.4239 | 0.5761 | 0.4112 | 0.5882 | 0.741 |  | 0.4239 | 0.5761 | 0.4274 | 0.5726 | 1.000 |  | 0.4239 | 0.5761 | 0.5053 | 0.4947 | 0.062 |
| rs2494251 (A/G) | 0.2709 | 0.7291 | 0.2783 | 0.7217 | 0.826 |  | 0.2709 | 0.7291 | 0.3145 | 0.6855 | 0.321 |  | 0.2709 | 0.7291 | 0.2606 | 0.7394 | 0.849 |
| rs11265165 (C/T) | 0.4388 | 0.5612 | 0.4433 | 0.5567 | 0.896 |  | 0.4388 | 0.5612 | 0.3548 | 0.6452 | 0.089 |  | 0.4388 | 0.5612 | 0.4579 | 0.5421 | 0.673 |
| rs12562171 (C/T) | 0.3687 | 0.6313 | 0.3695 | 0.6305 | 1.000 |  | 0.3687 | 0.6313 | 0.3065 | 0.6935 | 0.214 |  | 0.3687 | 0.6313 | 0.3989 | 0.6011 | 0.486 |
