# Supplementary material for: SNPs in the FCER1A Gene Region Show No Association with Allergic Rhinitis in a Han Chinese Population
Source: PLoS One. 2010 Dec 31;5(12):e15792. doi: 10.1371/journal.pone.0015792 (PMC3013135; doi:10.1371/journal.pone.0015792)
Supplement: Table S2 — Details of the primers used in the screening of SNPs by MassArray and PCR direct sequencing. (DOC) [file pone.0015792.s002.doc]

| SNP | Alleles | Gene | Function | Primers | Extension Primers |
| --- | --- | --- | --- | --- | --- |
| rs2494262 | A/C | / | Unknown | ACGTTGGATGCCAAGTCTCCTGAGATCATC  ACGTTGGATGAGCCTTCAGGTTCTACCTTC | CATCCTTTTGAGAATTCCT |
| rs2427836 | C/T | / | Unknown | ACGTTGGATGAGCACCACTGTTGAGAAACC  ACGTTGGATGGGATCAAGATTCAAATGAGC | AACGAGTTACAATCAAATGCAATA |
| rs2494263 | A/G | / | Unknown | TATTTTATCTGGGAACTCT ATGATAAAGGTTGATGGG | - |
| rs16841979 | C/G | / | Unknown | ACGTTGGATGTTTTATGACCGATGGCCTGC  ACGTTGGATGTATTCTGTGGTTAGAATTG | AAAGAGATGGACTTCTAATACT |
| rs2427837 | A/G | FCER1A | 5’ near gene | ACGTTGGATGCAGGTGCCCTCAAGGATTAT  ACGTTGGATGCTCTCTGTGTTACTACCTGG | TGCCCTCAAGGATTATTATAGT |
| rs12565775 | A/C | FCER1A | Intron | ACGTTGGATGACTTGGTTGATTTCTCCCCC  ACGTTGGATGGCTGTAATAACTGGGTAGAC | CCCAATTCTAGTACTTCCCTTAAAT |
| rs2494264 | A/T | FCER1A | Intron | ACGTTGGATGCAGTTTCAAAACGGAAAGAG  ACGTTGGATGTGGGCTAAGATCTCTCATCC | GAAATCTCTTAAAGGAATCCTTTT |
| rs11809585 | A/T | FCER1A | Intron | ACGTTGGATGACACAGTTGAATGCCACAGG  ACGTTGGATGAATTAGTAGCAGCCCACTCC | AAAAGTCCTTCAAGTTTCATA |
| rs2251746 | C/T | FCER1A | Intron | ACGTTGGATGAGGCACAGCTGATGGGTTAA  ACGTTGGATGCTGGAGAGATCTAAGGCTTC | ATGGGTTAACCAGATATGA |
| rs2298804 | A/G | FCER1A | Missense | ACGTTGGATGCTTGTTGGTGCTGACATTTG  ACGTTGGATGATGGCAGCCTTTCAGAAGAG | TTCTCCACTGTCTTCAAAT |
| rs2298805 | A/G | FCER1A | Missense | ACGTTGGATGCCATATCCCTGGAACTTACC  ACGTTGGATGCAAATGTCAGCACCAACAAG | CTTCCAGGTACACAGGTTCA |
| rs2494265 | A/T | FCER1A | Intron | ACGTTGGATGCTTTATTTAATGTCCTCTTC  ACGTTGGATGGAATGTAGAACTAGACAGGG | TTTAATGTCCTCTTCCCATAC |
| rs2269718 | A/G | FCER1A | Intron | ACGTTGGATGGAGAGAGAGAATGAATAGAG  ACGTTGGATGTCATAGTTTCTGACACATGC | AGAATGAATAGAGAAAAGAGAG |
| rs2494251 | A/G | / | Unknown | ACGTTGGATGGACTTCCTATGGATTCTGAG  ACGTTGGATGGGCAGCAGTGCAGTATTATC | AAGTTCAGATTTTAATTTTTTAACCA |
| rs11265165 | C/T | OR10J3 | Missense | ACGTTGGATGTGGATGATGACCACTGTGAG  ACGTTGGATGCCTCATCATCTCCACCATTC | TGGCAAAGGCCTTCTTC |
| rs12562171 | C/T | / | Unknown | ACGTTGGATGCAGTGCCTCCTGAAATTCTC  ACGTTGGATGAATGGCAGTCAGTAGTTGGG | CTTCCTGTCCCCTCCAC |
